# Knowledge, attitude and practices on intermittent preventive treatment in pregnant women with malaria: a mixed method facility-based study in Western Kenya

**DOI:** 10.11604/pamj.2024.48.22.42196

**Published:** 2024-05-28

**Authors:** Joseph Mukala, Dominic Mogere, Peter Kirira, Bernard Kanoi, Violet Akisa, Francis Kobia, Harrison Waweru, Jesse Gitaka

**Affiliations:** 1 School of Public Health, Mount Kenya University, Thika, Kenya,; 2 School of Applied Sciences, Mount Kenya University, Thika, Kenya,; 3 Centre for Malaria Elimination, Institute of Tropical Medicine, Mount Kenya University, Thika, Kenya,; 4 Webuye County Hospital, Webuye, Kenya

**Keywords:** Knowledge, attitude, practices, pregnancy, malaria

## Abstract

**Introduction:**

intermittent preventive treatment remains a core strategy for malaria prevention in pregnancy. Sulfadoxine-pyrimethamine is recommended for all pregnant women in malaria-prone zones. It is scheduled monthly at each antenatal care visit for up to 36 weeks. Here, we sought to assess the knowledge, attitude, and practices of intermittent preventive treatment among pregnant women with malaria in Webuye Hospital.

**Methods:**

a total of 140 participants aged between 18 and 49 years and at approximately 16 weeks of gestation were enrolled in this study, which utilized a mixed qualitative-quantitative method. Before enrollment, malaria testing was conducted using microscopy, and participants were divided into two cohorts: malaria-positive and malaria-negative. Close-ended and open-ended questionnaires were used. Qualitative-quantitative data analyses were performed.

**Results:**

our analysis revealed a significant difference between the proportion of mothers in the negative and positive groups in terms of their knowledge about side effects (p ≤ 0.001) and different doses (p ≤ 0.012) of intermittent preventive treatment. The proportion of mothers who knew side effects and different doses was higher among the malaria-positive group as compared to malaria-negative group with 37(52.9%, n=70) versus 18(25.7%, n=70) and 14(20.0%, n=70) versus 4(5.7%, n=70) respectively. Additionally, there was also a significant difference in knowledge about intermittent preventive treatment before administration (p ≤ 0.003) between the two groups.

**Conclusion:**

good knowledge, attitude and practices on intermittent preventive treatment (IPT) benefits, side effects, safety, doses and other prior information should be leveraged to empower pregnant women in malaria-endemic zones.

## Introduction

Globally, malaria infection is a serious communicable infectious disease that threatens the life of the half of world's population with approximately 515 million people in Latin America, Asia, and sub-Saharan Africa region with one to three million deaths each year [1]. Recently, malaria has affected 228 million people worldwide, with approximately 213 million in sub-Saharan Africa representing 93% of the total population. The most vulnerable persons are children and expectant women with consequences ranging from deadly complications such as anemia, abortion, intrauterine fetal retardation, small gestation for age, prematurity and low birth weight. Indeed, Sulfadoxine-pyrimethamine is a strongly recommended molecule for all pregnant women in moderate to high malaria transmission zones except HIV patients, and Kenya adopted three doses that are efficient and efficacious to protect pregnancy against malaria [2,3].

The utilization and uptake of highly cost-effective interventions for malaria were found to be associated with poor maternal knowledge, complicated guidelines, and policy issues barring healthcare workers from delivering efficient routine antenatal care. However, different barriers to accessing these interventions are poorly investigated in the current context. Therefore, innovation in terms of malaria identification, eradication, and diagnostic strategies is needed [4]. Despite the recommendation for pregnant women living in endemic malaria zones to receive sulfadoxine-pyrimethamine (SP), Kenya presented sub-optimal uptake of intermittent preventive treatment estimated at 13% of those who received three or more doses, while 28% received one or more doses. However, Bungoma County recorded 45% of pregnant women who received more than three doses which still is below the universal coverage [5].

The Malaria Strategy Plan for 2019-2023 had set its goal at 100% coverage of all people at risk residing in malaria-prone zones by providing them with access to effective malaria preventive interventions. In Kenya, the increase of utilization was fixed at least at 80 percent by 2023 [6]. World Health Organization established a malaria-free world vision stating that countries should take opportunities to innovate local and adapted activities in line with global recommendations such as equity in access to health services especially for the most vulnerable and hard-to-reach populations. Therefore, this goal translates into actions which can reduce malaria incidence and mortality gradually from 90% to 40% between 2020 up to 2030 [7]. A multiple indicator cluster survey carried out among pregnant women in Bungoma County, which is one of the most endemic malaria areas in the Western Kenya region, highlighted that out of 57% of the population who slept under the mosquito nets, 70% of them were pregnant, 22% only had received 2 doses and plus of intermittent preventive treatment. In addition, the survey stated that there were 31 deaths among children of less than five years over 1000 live births against a recorded national average of 22 deaths over 1000 live births which should be addressed to fill the gap [8-10].

The County Government of Bungoma listed malaria and anemia as the most frequent disease burden in its integrated development plan 2018-22 and targeted to bridge the existing gap by improving the low rate of maternal and infant indicators through the offer of quality antenatal care service to the vulnerable population. It was established that the half of pregnant women in the county had not achieved antenatal clinic four visits as recommended by the WHO guideline [11]. Roll Back Malaria partnership emphasizes that antenatal care should be an entry point where every pregnant woman will be served with at least one dose of Fansidar and at least 80% will receive insecticide-treated nets with an ambition to reach 100% by 2025 [12].

Previous studies have shown numerous benefits associated with the use of intermittent preventive treatment (IPT) with regard to knowledge of expectant women, but little is known of what pregnant women knew and translated into actionable “practices”. The explicit inclusion of qualitative opinions and views from the main beneficiaries regarding the benefits, side effects, schedule, doses, safety, prior information, attitude of healthcare providers, trust, and sources of information can enhance uptake and the utilization of intermittent preventive treatment.

**Objectives of the study:** to assess knowledge, attitude, and practices on intermittent preventive treatment among pregnant women with malaria in Webuye Hospital, Bungoma County.

**Research questions:** how do knowledge, attitudes and practices of pregnant women contribute to improve intermittent preventive treatment in Webuye hospital, Bungoma County?

## Methods

**Study design:** this is a qualitative-quantitative method study with variables drawn from a prospective cohort research study conducted in Webuye Hospital from (March 2022) to (December 2022). Before enrolment, a key condition was malaria test results to ensure that pregnant women had malaria or not. This approach was used until the number of subjects was reached in each arm. A follow-up was thereafter conducted until delivery.

**Setting:** this study participants were subdivided into two arms; arm 1 comprised malaria-positive, while arm 2 comprised malaria-negative pregnant women conducted in Bungoma County, Webuye Hospital. The County has a population estimated at 1,919,490 with 939,105 males and 980,385 females, and 429,762 women of childbearing age 15-49 years. The County covers an area of 3032 km^2^ and lies between latitude 00 28´ and latitude 10 30´ North of the Equator, and longitudinal 340 20´ East and 350 15´ East of the Greenwich meridian [13]. It borders the Republic of Uganda to the Northwest, Trans-Nzoia County to the Northeast, Kakamega County to the East and South-East, and Busia. There are two rainy seasons, the long rainy season goes from March to July, and a short season from August to October with an annual rainfall ranging from 400 mm to 1,800 mm. The temperature varies between 0 celsius and 32 celsius [11].

**Participants:** the study targeted pregnant women, attending the antenatal clinic at Webuye Hospital, aged between 18 and 49 years from 16 weeks of gestation. At this gestational age, pregnant women were safe to receive intermittent preventive treatment during routine antenatal clinics in the rural setup.

**Study size:** the sample size calculation formula for the cohort was used based on malaria prevalence in the non-exposed group estimated at 28% according to the study of Nyamu [14]. The prevalence of malaria in the exposed group was estimated at 6.1% according to the demographic health information system 2 [13]. Beta (10%), alpha (5%), confidence level of 95%, Z alpha (1.96), Z beta value (1.28), sample size for group-1 (n1=60), sample size for each group-2 (n2=60), sample size for both group (n1+n2=120), attrition (=20%), total sample size with attrition=144.

**Sampling frame and criteria:** participants were pregnant women attending the antenatal clinic at Webuye Hospital, Bungoma County. Only pregnant women from 16 weeks gestation and who were residing in the area for almost six months were enrolled in one of the two corresponding arms until 72 participants were reached in each arm. The study was conducted during a period from (March 2022) to (December 2022). These participants were targeted in an ongoing prospective cohort study aimed at determining birth weight at delivery among pregnant women and the condition for enrollment was a malaria test. When malaria was positive participant was systematically enrolled as exposed (arm 1).

**Data collection:** assistant researchers were trained, and open and close-ended questionnaires were pretested in the mother-child health service two weeks before the study. Twenty pre-selected pregnant women who attended antenatal care services accepted to give their written consent in the survey. During the session corrections were made and a final English and Kiswahili version was adopted and validated with supervisors´ approval. During the data collection process answers were provided by participants and incomplete response were cleaned before data analysis process. Two focus group discussions were organized, each lasting 60 minutes to collect views and insights of pregnant women on intermittent preventive treatment while notes, images, and video records were taken.

**Statistical analysis:** answers were written, cleaned from field errors, coded, and fed into SPSS 27. Knowledge of intermittent preventive treatment with different variables were captured through the responses yes or no. Variables were benefits, side effects, doses, schedule, safety, prior information on the medicine, sources of information, and malaria tests were analyzed using Chi-square of association at p-value <0.05 (95%) and percentage. Attitudes included variables such as trusting information given by healthcare workers, the attitude of healthcare workers during the administration of intermittent preventive treatment-sulfadoxine-pyrimethamine (IPT-SP) and awareness of the benefits of taking intermittent preventive treatment analyzed by the means of the Likert scale and percentage. Practices included having received one dose of IPT/SP, two doses, three doses or none. Data collected were analyzed by means of the computer-assisted qualitative data analysis (MAXQDA), percentage, and Chi-square of the association at a p-value <0.05 (95%). The third dose of IPT/SP was considered in practice as a complete dose. The results were presented in the form of tables and figures. Themes and sub-themes were developed during two focus group discussions and captured in the form of in-depth views and opinions among positive and negative malaria cohorts, as well as video recording, transcribing, coding and analyzing using computer-assisted qualitative data analysis software (MAXQDA). Chi-square test of association was computed to test the statistical significance at a p-value equal to or less than 0.05 (95%).

**Focus group discussion description:** qualitative variables were organized into two themes whereby the first was focused on the knowledge and attitude of the intermittent preventive treatment with different subthemes: the reason for use of IPT/SP in pregnancy, number of intermittent preventive treatment doses should a pregnant women receive during pregnancy, interval between one dose to another. The second concerned practices of preventing malaria during pregnancy comprising the following subthemes: what pregnant women do practically to prevent malaria during pregnancy. Two focus group discussions were organized with 6 participants each to collect the views of pregnant women on intermittent preventive treatment. Their statements were captured below as part of qualitative speech indicating in-depth views and opinions of what they knew and practiced using MAXQDA software using the following speeches: “*I am sure it is almost two times I have been given this medicine when going to the antenatal clinic, I took it with water after the nurse explained it to us and I have never refused to swallow it*”. Mother two focus group discussion one. “*The nurse told me that it is very paramount to take each month until I will be close to delivery for me to stop malaria disturbance I am going regularly to the clinic and I received one dose so far*”. Mother three focus group discussion one. “*I am confident that I took three times the tablets somehow they were not friendly but the nurse explained clearly that it was to curb malaria infection*”. Mother four focus group discussion one “*I swallowed three doses each time I went to clinic the nurse insisted on immediate swallowing in the hospital although there were some challenges*”. Mother five focus group discussion one. “*I had taken it three times and feel nauseated, the nurse was very supportive saying it is a good way to tackle malaria*”. Mother six focus group discussion one. “*I swallowed one dose of three tablets and was encouraged to be continuously coming to take, I did not find any difficulty since the explanation was clear*”. Mother one focus group discussion two. “*I got two doses and decided to end their due to the side effects*”. Mother two focus group discussion two. “*I can confirm that I took two doses but did not find any reason to be right since I also use folic acid*”. Mother five focus group discussion two. “*I cannot tell with exactitude how many doses so far but think had gotten twice. Every time “I go for an antenatal visit I have been explained and swallowed on the spot two consecutive times and I have never refused to take the medicine*”. Mother one focus group discussion one. “*I am sure to have used three doses when attending the clinic, it is making me strong and my baby will be safe*”. Mother six focus group discussion two.

**Data source/measurement/bias:** the pretesting of questionnaires was conducted in the mother-child health service two weeks before the study began. Supervisors, assistant researchers, and respondent´s insights during the discussion were taken in consideration before validation of questionnaires. Close and open-ended questionnaires addressed quantitative aspects whereas focus group discussion was used for qualitative aspects. Video record materials were transcribed and coded before analysis. The computation for quantitative data was analyzed using percentage and Chi-square whereas qualitative data was analyzed using Likert-scale, participant verbal speech, and percentage.

**Ethical considerations:** ethical approval was sought from the Ethics Review Committee of Mount Kenya University, and a research permit was obtained from NACOSTI (MKU/ERC/2100, license No. NACOSTI/P/22/16233), as well as the local authorizations obtained from County and Webuye Hospital. Each respondent was explained prior to signing written informed consent. In addition, the explanation concerned the respondent´s rights, confidentiality and anonymous character of data generated, voluntary participation, respect, withdrawal at any time, compensation, and discard of video tape and images at the end of the study.

## Results

The key findings of this study are presented based on 140 (100%) participants subdivided into malaria-positive and malaria-negative cohorts. Knowledge of intermittent preventive treatment comprised variables such as benefits, side effects, doses, schedule, safety, prior information on the medicine, sources of information, and malaria tests were analyzed using Chi-square of association at p-value <0.05 (95%) and percentage. Attitudes include variables such as trusting information given by healthcare workers, the attitude of healthcare workers during the administration of IPT/SP, and awareness of the benefits of taking intermittent preventive treatment analyzed under Likert-scale and percentage. Practices include having received one dose of IPT/SP, two doses, three doses or none was analyzed under percentage and Chi-square of association at a p-value<0.05 (95%). The third dose of IPT/SP was considered in practice as a complete dose.

**Figure 1:**
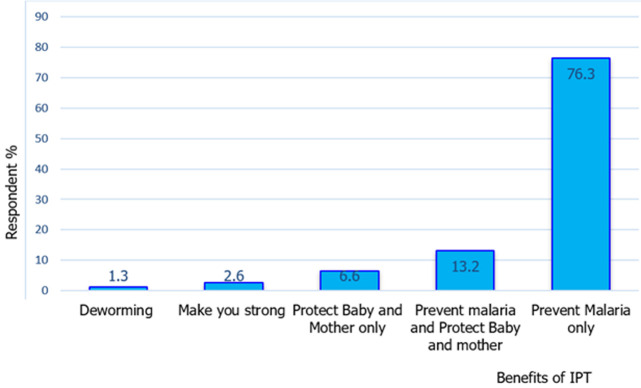
benefits of intermittent preventive treatment (IPT)

**Knowledge of intermittent preventive treatment:** the findings demonstrated that 63 (45%) respondents did not know the benefit of respondents whereas 77 (55%) knew the benefit of IPT. In both groups, there was no difference in p-value=0.610. In addition, there was a significant difference in the proportion of negative and positive malaria groups among mothers´ knowledge of the side effects (p-value=0.001), different doses (p-value=0.012), and those who were informed about intermittent preventive treatment before administration (p-value=0.003), The proportion of mothers knowledge about side effects and different doses were higher among the malaria positive group as compared to the negative group (52.9% versus 25.7% and 20.0% versus 5.7%) respectively. However, only half of the malaria-positive group were informed about the intermittent preventive treatment of sulfadoxine-pyrimethamine before it was administered as compare to 74.3% in the negative group (Table 1). Furthermore, emphasizing knowledge of intermittent preventive treatment, the study revealed that concerning the benefits of using IPT/SP among pregnant women who were interviewed, 1.3% of respondents cited that it was for deworming, 75 (76.3%) reported that IPT/SP prevents malaria, followed by 24 (13.2%) who reported that it prevents malaria and protects baby and mother (Figure 1).

**Table 1:** intermittent preventive treatment

		Malaria test		
**Variables**	**Overall, N = 140**	**Negative, n = 70**	**Positive, n = 70**	**P-value**
**Benefits of IPT, n (%)**				0.610
No	63 (45.0)	33 (47.1)	30 (42.9)	
Yes	77 (55.0)	37 (52.9)	40 (57.1)	
Knowledge of side effects of IPT, n (%)	55 (39.3)	18 (25.7)	37 (52.9)	0.001
Different doses of IPT, n (%)	18 (12.9)	4 (5.7)	14 (20.0)	0.012
Safe drug during the first trimester, n (%)	83 (59.3)	47 (67.1)	36 (51.4)	0.058
Ever refused to take IPT, n (%)	20 (14.3)	13 (18.6)	7 (10.0)	0.150
Have you been informed about IPT, n (%)	87 (62.1)	52 (74.3)	35 (50.0)	0.003
Trust information given by HCW, n (%)	124 (88.6)	65 (92.9)	59 (84.3)	0.110
**Attitude of HCW towards provision, n (%)**				0.300
Bad	21 (15)	9 (12.9)	12 (17.1)	
Good	49 (34.9)	27 (38.5)	22 (31.4)	
Very good	70 (49.9)	34 (48.5)	36 (51.4)	
**IPT doses given, n (%)**				<0.001
None	29 (20.7)	24 (34.3)	5 (7.1)	
Once	34 (24.3)	17 (24.3)	17 (24.3)	
Twice	46 (32.9)	11 (15.7)	35 (50.0)	
Thrice	31 (22.1)	18 (25.7)	13 (18.6)	

N= total number of the study respondents; n= number of respondents in a given cohort; the Chi-square of association was used at p-value <0.05 (95%); IPT= intermittent preventive treatment; HCW= healthcare workers

**Figure 2:**
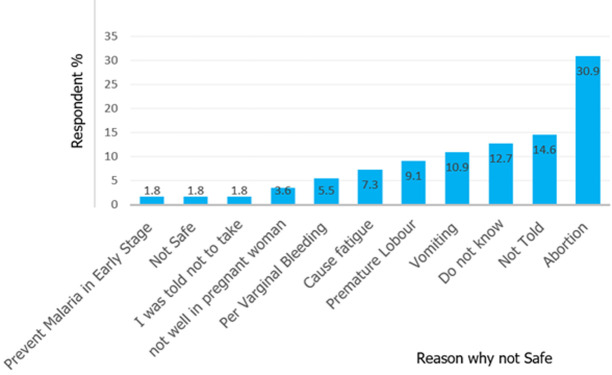
reported reasons of not using intermittent preventive treatment/sulfadoxine-pyrimethamine (IPT/SP) during the first trimester

**Attitudes towards intermittent preventive treatment:** the findings demonstrated that the majority of pregnant women 70 (49.9%) reported that healthcare workers (HCWs) had good attitudes towards the provision of the service. 124 (88.6%) respondents trusted the information given by healthcare workers (Table 1). More than half of respondents 7(58%) were aware of the benefit of sulfadoxine-pyrimethamine against 5 (42%). Pregnant women expressed that sulfadoxine-pyrimethamine prevents malaria and those aware were 7 (58%) (Table 2). Emphasizing the attitude of respondents the study explored the reason for not using IPT/SP during the first trimester among the 140 participants interviewed, 30.9% had the opinion that it causes abortion, 10.9% said it causes vomiting, 9.1% premature labor and 7.3% said it causes fatigue as well as other reasons (Figure 2).

**Table 2:** benefits of sulfadoxine-pyrimethamine

Knowledge of the benefits of IPT-SP	Frequency	Percentage	Cumulative
Aware	7	58%	58%
Unaware	5	42%	100%
Total	12	100 %	100%

Percentage was used to calculate knowledge awareness among participants; intermittent preventive treatment/sulfadoxine-pyrimethamine (IPT/SP)

**Figure 3:**
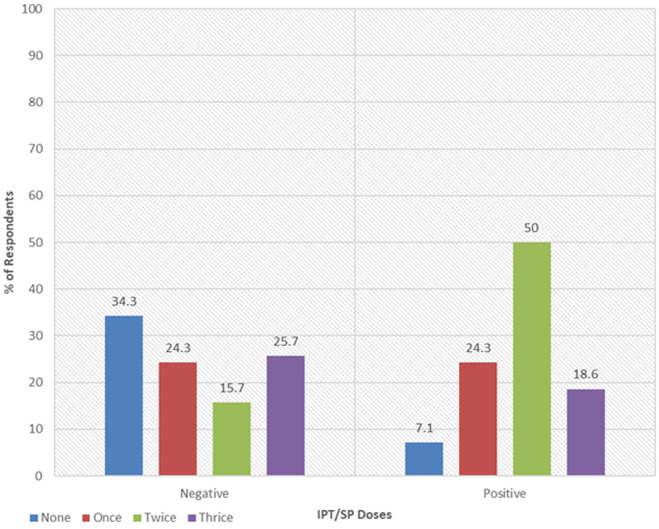
intermittent preventive treatment-sulfadoxine-pyrimethamine (IPT -SP) doses received

**Practices of intermittent preventive treatment among respondents:** there was a statistical association between the number of IPT/SP doses and malaria tests (p-value<0.001). Overall, most women received two doses of IPT (n = 46; 32.9%). Among the malaria-negative cohort, 24 (34.3%) did not receive any dose while 11 (15.7%) received two doses. This was different with malaria-positive cohort 35 (50.0%) who received two doses of IPT/SP and only 5 (7.1%) had not received any dose (none). The total coverage of IPT/SP 2 doses was higher among malaria-positive pregnant women 48 (68.6%) than among malaria-negative pregnant women 29 (41.4%) (Table 1). Focus group discussions were organized to collect pregnant women's opinions as well as their views, which were captured, recorded, transcribed, then coded and analyzed by the mean of MAXQDA Software analysis to identify practices concerning the completeness of the sulfadoxine-pyrimethamine during pregnancy. The results showed that 10 (83%) pregnant women had completed IPT/SP doses which is considered as a key element toward malaria prevention during pregnancy (Table 3). Emphasizing the practices, the study revealed that the cohort of malaria positive respondents 35 (50%) received 2 doses of IPT/SP compared to 11 (15.7%) in the cohort of malaria-negative respondents. Those who received 3 doses were 18 (25.7%) in negative cohort versus 13 (18.6%) in positive cohort (Figure 3).

**Table 3:** practices of intermittent preventive treatment

Sulfadoxine-pyrimethamine	Frequency	Percentage
Practicing/completing	10	83%
No practicing/refusing	2	17%
**Total**	**12**	**100%**

Views of respondents expressed in percentage concerning the completeness of intermittent preventive treatment-sulfadoxine-pyrimethamine (IPT-SP)

## Discussion

In a research survey done in Sabatia Kenya under a cross-sectional design, it was found that a good number of pregnant women had good knowledge of the benefit of intermittent preventive treatment with sulfadoxine-pyrimethamine benefits, but did not know the exact time for the beginning of intermittent preventive treatment/sulfadoxine-pyrimethamine and never experienced sulfadoxine-pyrimethamine side effects [15]. However, this study found that marital status, knowledge of the benefits of sulfadoxine-pyrimethamine, and gestation age were significantly associated with uptake of sulfadoxine-pyrimethamine, with women who had good knowledge of benefits having a higher likelihood of receiving the third dose than those with poor knowledge. In our study, we found that the two key variables which determined the completeness of IPT/SP were practices and malaria tests. The first was found to have been associated with completeness through qualitative opinion in that pregnant women with good practices had this to say ”I had finished 2 or 3 doses, “ whereas the second factor showed that pregnant women with positive malaria tests had a higher likelihood to complete the second dose as compared to those with negative malaria test. A research survey under a qualitative research design conducted in two countries; Mali and Kenya using focus group discussion, found that despite having correct knowledge of doses and IPT/SP intake intervals, expectant women felt that the method was still very powerful to be avoided during pregnancy due to possible induction of miscarriage [16].

The result of our study found that 30% of participants had the same opinion that IPT/SP causes abortion. Strikingly, the safety variable as an important parameter on the side of pregnant women and their unborn infants was raised as a reason why pregnant women do not accept IPT/SP during the first trimester but also for the subsequent doses. Similarly, the same concern was raised in the research study conducted in Somalia [17]. We found that the result of our study was in agreement with a qualitative study carried out in Mozambique citing the word of a pregnant woman going to the antenatal clinic who recognized sulfadoxine-pyrimethamine tablets as the white tablets given to pregnant women and took in the presence of healthcare worker. Saying that “*I was not told more but I was given three tablets which I took”. Strikingly, she argued that “Some pregnant women even when advised and counseled do not complete the doses of sulfadoxine-pyrimethamine*” (maternal and child nurse) [18].

A descriptive research design found that approximately all respondents had heard about IPT/SP and 57% stated that this medicine was convenient with malaria prevention in both mothers and unborn children, and 15.4% felt that it was used to treat malaria [19]. However, nausea, vomiting, body weakness, headache, dizziness, abdominal pain, and diarrhea were reported as unwanted effects of the molecule used to prevent malaria in expectant women. This previous finding corroborates the finding of our study which showed that these unwanted side effects did not prevent pregnant women from receiving the subsequent doses of IPT/SP. Therefore, we found that knowledge of pregnant women vis-a-vis IPT/SP benefits, schedule, doses, and side effects was acceptable and can be translated into good practices as demonstrated in the qualitative insight. We are in support of the following studies which were carried out in Uganda and Nigeria. According to their findings, the Ugandan study laid down the credence that attendance at the antenatal clinic increased the accessibility to the IPT/SP [20], while the Nigerian study argued that all pregnant women do not attend the clinic session during pregnancy [21]. Therefore, giving room to support the work of healthcare providers´ prior information given to pregnant women to empower them on IPT/SP, which can translate later into acceptance and completeness of the medicine used to prevent malaria, beyond laying a good ground for community strategies targeting healthcare workers to reach every pregnant woman [21,22].

**Strengths of the study:** the combination of quantitative and qualitative results sound as an appropriate approach to give a more insightful “knowing and doing” towards intermittent preventive treatment as a cost-effective strategy to improve mother-child health outcomes in endemic malaria-prone areas.

## Conclusion

Good knowledge, attitude and practices of intermittent preventive treatment on side effects, doses and prior information constitute a cost-effective strategy, which should be further leveraged to empower pregnant women in malaria-endemic areas toward universal goals achievement.

### What is known about this topic

- *Malaria preventive strategy for pregnant women living in the endemic zones relies mainly on the administration of intermittent preventive treatment to every pregnant woman;*
- *Pregnant women in the Western Kenya region are at high risk of malaria and are more likely to be protected when they are given repetitive doses of intermittent preventive treatment besides other methods*.

### What this study adds

- *This study uses qualitative-quantitative data intended to clarify what pregnant women know and practice at the same time to fulfill intermittent preventive treatment strategy; therefore, pregnant women who had malaria in their life were more likely to report more side effects due to the sulfadoxine-pyrimethamine use due to the exposure than those who have not used this medicine and not being exposed to malaria before;*
- *The proportion of mothers’ knowledge about side effects, prior information, and different doses of intermittent preventive treatment was found higher among the malaria-positive group as compared to the negative group meaning that pregnant women who have suffered from the disease have increased exposure to reliable information through their contact with healthcare providers*.
